# Correction to: ‘Phylogenomics of Aplacophora (Mollusca, Aculifera) and a solenogaster without a foot’ (2019) by Kocot *et al.*

**DOI:** 10.1098/rspb.2022.2057

**Published:** 2022-11-09

**Authors:** Kevin M. Kocot, Christiane Todt, Nina T. Mikkelsen, Kenneth M. Halanych

*Proc. R. Soc. B* **286**, 20190115. (Published online 8 May 2019) (https://doi.org/10.1098/rspb.2019.0115)

In this study, we investigated evolutionary relationships within Aplacophora using transcriptome data. Additional morphological examinations and DNA barcoding have revealed that two specimens sequenced in this study were mis-identified by K.M.K. The specimen identified as *Entonomenia tricarinata* (Rhopalomeniidae) has been determined to be *Dorymenia tricarinata* (Proneomeniidae), which has a similar habitus and overlapping distribution. The specimen identified as *Meiomenia swedmarki* (Meiomeniidae) has been determined to be an undescribed species belonging to the same order (Pholidoskepia) that is superficially similar in external appearance and occurs at the type locality of *M. swedmarki*. Correcting these errors does not alter the main conclusions of the original paper. However, it does provide evidence that the family Proneomeniidae (represented by *Dorymenia* and *Proneomenia*) is not monophyletic as *Dorymenia tricarinata* was recovered as the sister taxon of *Epimenia babai* (Epimeniidae) rather than the two sampled *Proneomenia* species. The organism field has been corrected for these transcriptomes on NCBI SRA (see accession numbers SRX3872458 and SRX3872469).

