# Supplementary figures and images for: Phylogenomics of Aplacophora (Mollusca, Aculifera) and a solenogaster without a foot
Source: Proc Biol Sci. 2019 May 8;286(1902):20190115. doi: 10.1098/rspb.2019.0115 (PMC6532501; doi:10.1098/rspb.2019.0115)

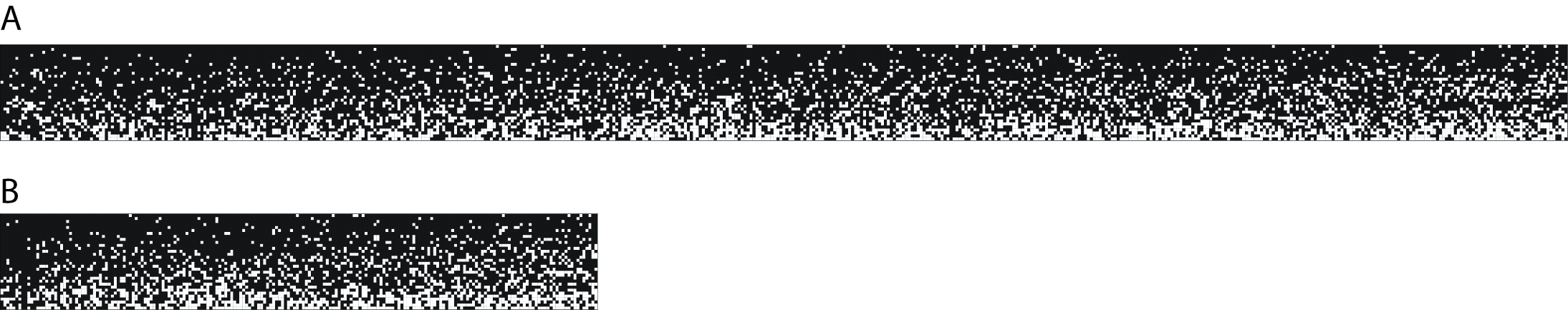

Supplement: Supplementary Figure 1 [file rspb20190115supp1.tif]

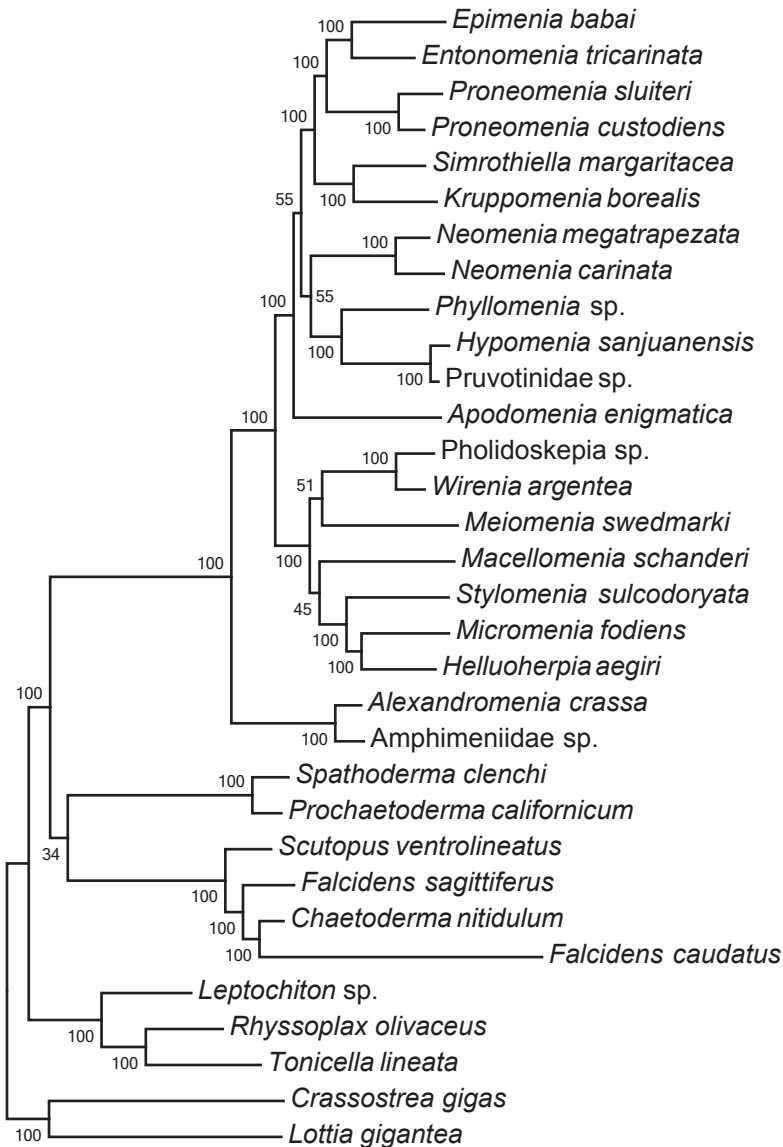

Supplement: Supplementary Figure 2 [file rspb20190115supp2.pdf]

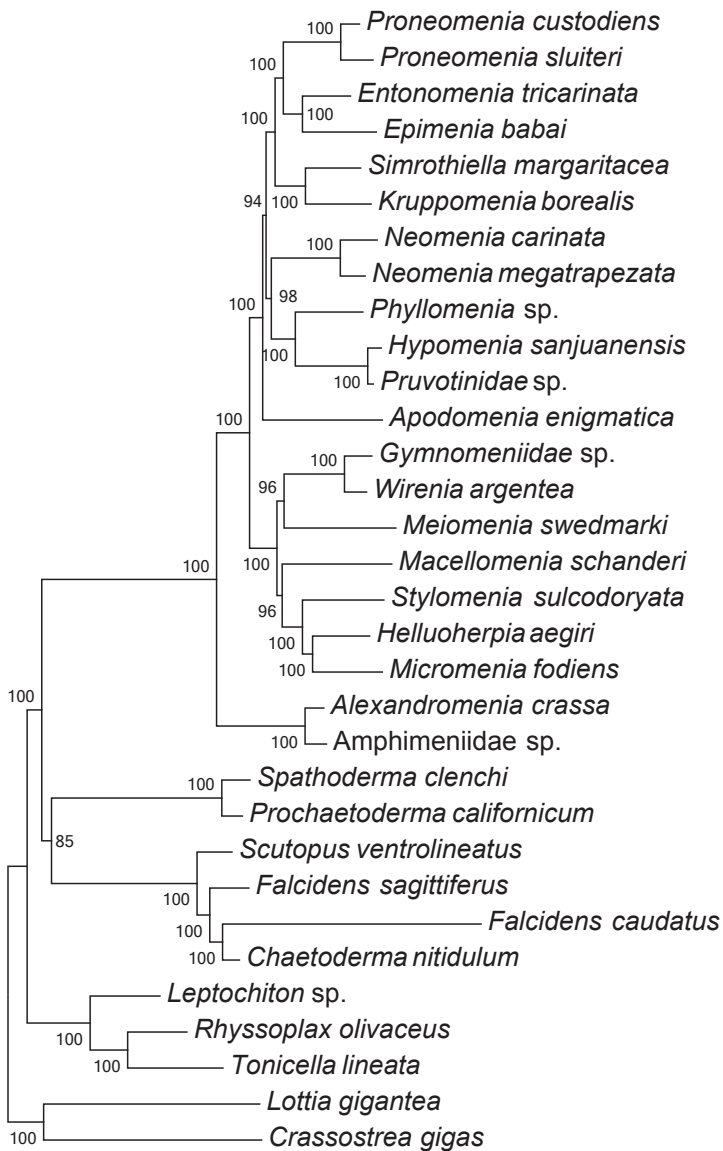

Supplement: Supplementary Figure 3 [file rspb20190115supp3.pdf]

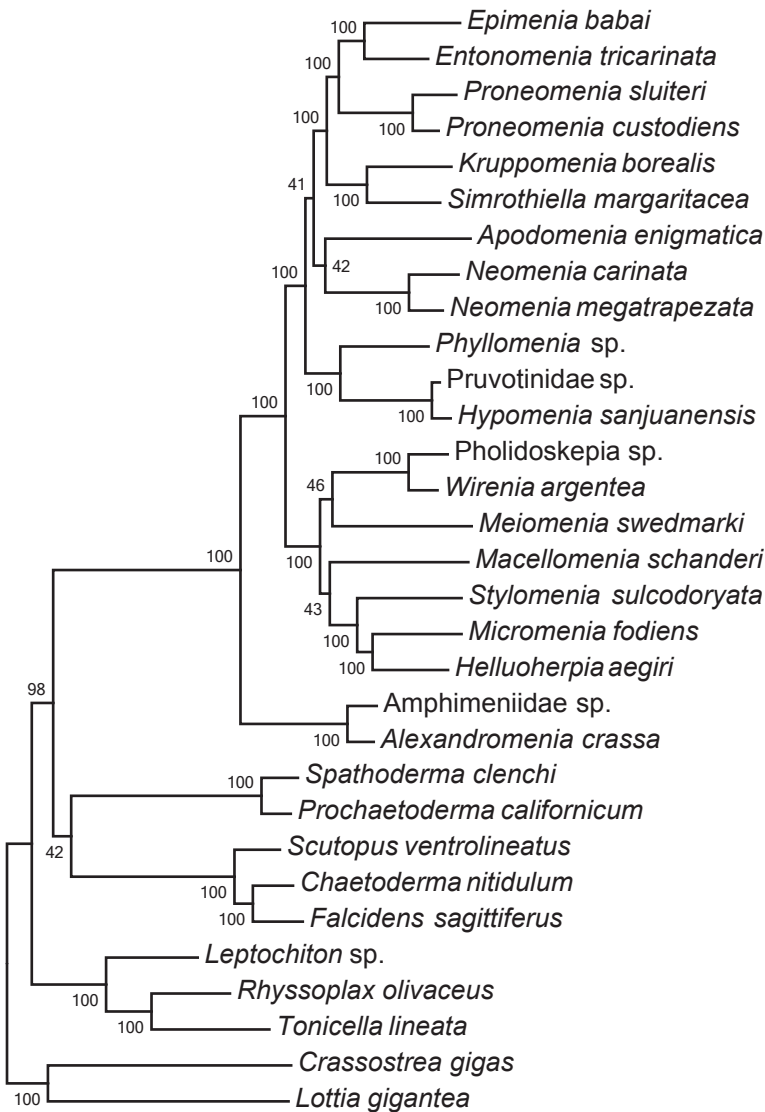

Supplement: Supplementary Figure 4 [file rspb20190115supp4.pdf]

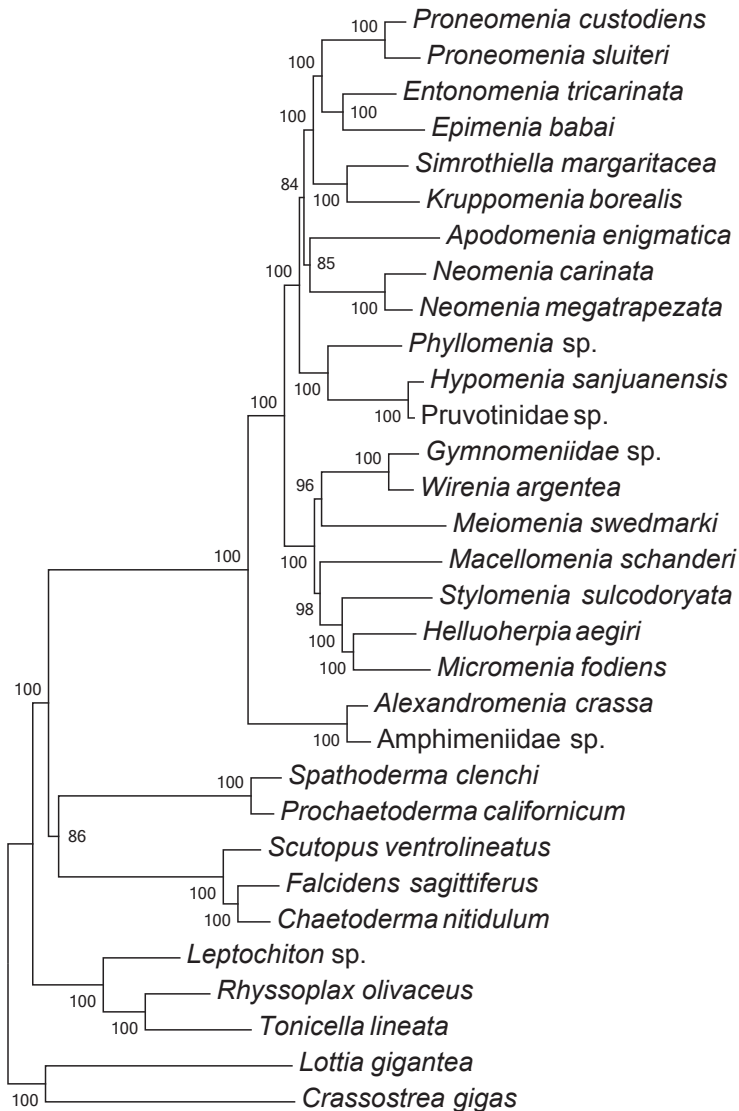

Supplement: Supplementary Figure 5 [file rspb20190115supp5.pdf]

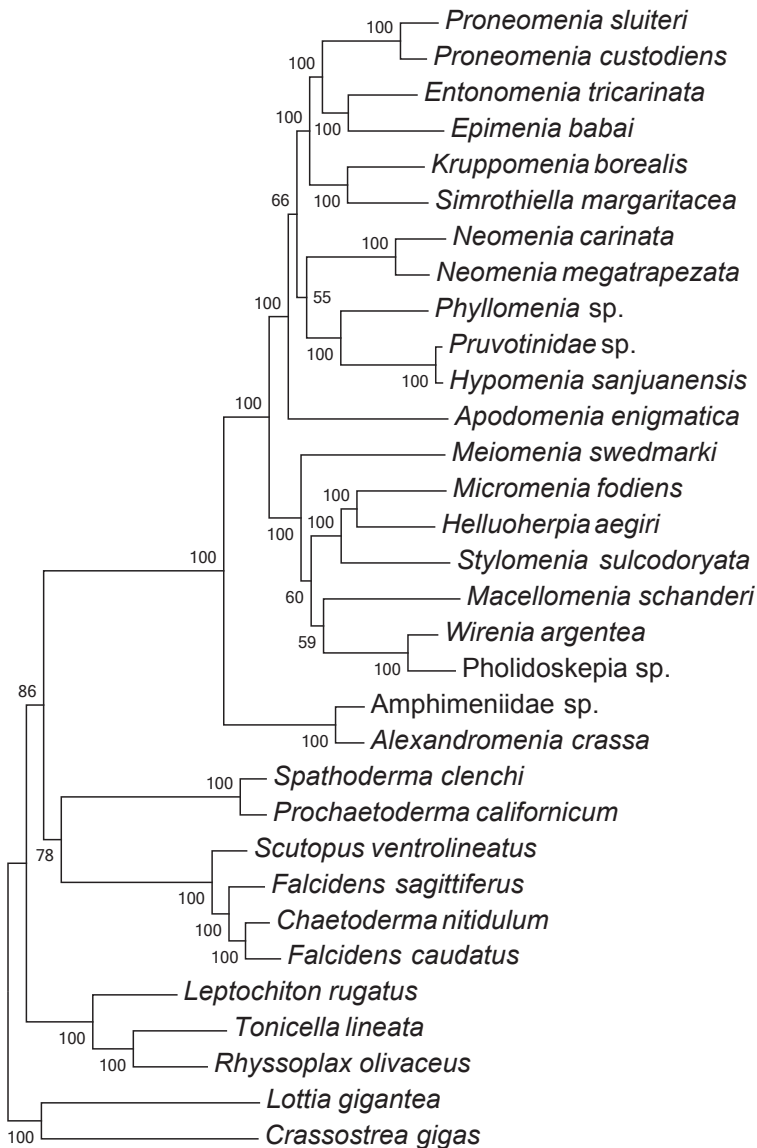

Supplement: Supplementary Figure 6 [file rspb20190115supp6.pdf]

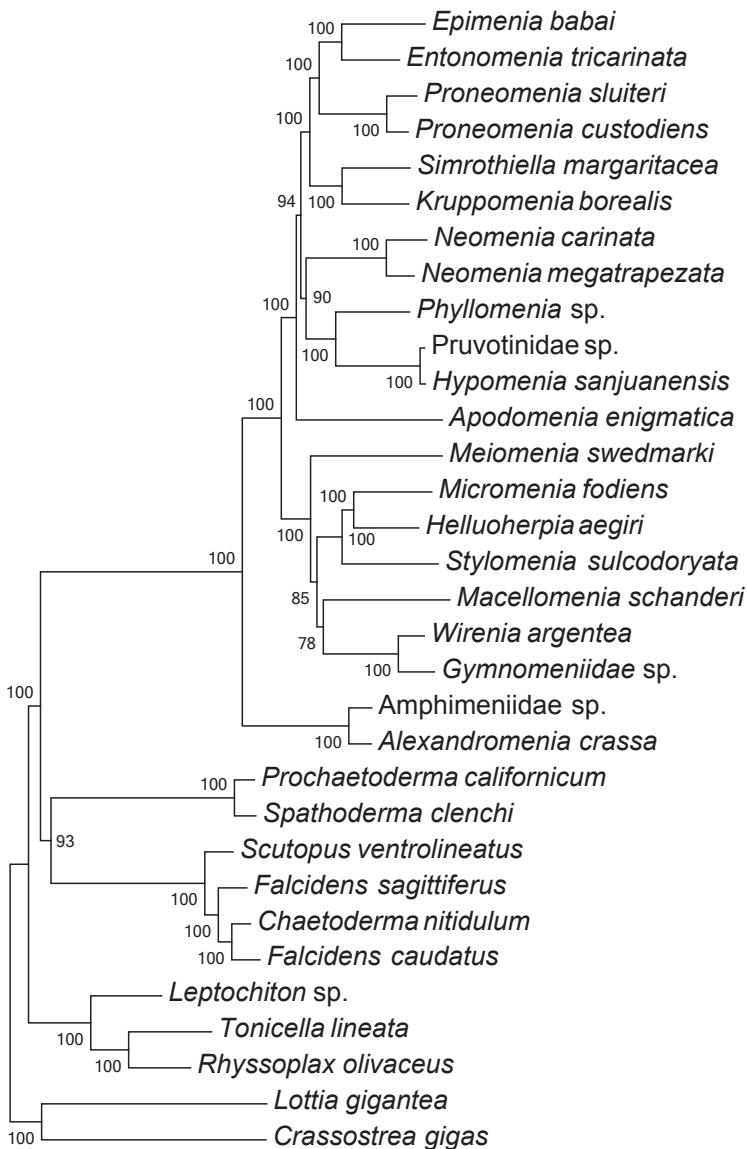

Supplement: Supplementary Figure 7 [file rspb20190115supp7.pdf]
