# Supplementary material for: Phylogenomics of Aplacophora (Mollusca, Aculifera) and a solenogaster without a foot
Source: Proc Biol Sci. 2019 May 8;286(1902):20190115. doi: 10.1098/rspb.2019.0115 (PMC6532501; doi:10.1098/rspb.2019.0115)
Supplement: Supplementary Figure 8 [file rspb20190115supp8.pdf]

```
                       10        20        30        40        50        60        70        80        90       100
              ....|....|....|....|....|....|....|....|....|....|....|....|....|....|....|....|....|....|....|....|
transcriptome TTGCTGTTTAGAATATGGGCCGGTATAGTTGGTGCCGGCCTAAGAATATTAATTCGAATTGAACTGGGTCAGCCTGGGCCAATTTTCGGGGATGATCACC
PS96_310R     ..............G.................C.........................................C.........................
Ap199.3C      ....................................................................................................
Ap200.2E      ....................................................................................................
Ap225.2C      ....................................................................................................
Ap233.1E      ....................................................................................................

                      110       120       130       140       150       160       170       180       190       200
              ....|....|....|....|....|....|....|....|....|....|....|....|....|....|....|....|....|....|....|....|
transcriptome TATACAATGTAATTGTTACTGCTCATGCGTTTGTAATAATTTTTTTTTTGGTGATGCCTATAATAATAGGGGGTTTTGGTAACTGATTAATTCCGTTAAT
PS96_310R     ....T.................C.............................................................................
Ap199.3C      ....................................................................................................
Ap200.2E      ....................................................................................................
Ap225.2C      ....................................................................................................
Ap233.1E      ....................................................................................................

                      210       220       230       240       250       260       270       280       290       300
              ....|....|....|....|....|....|....|....|....|....|....|....|....|....|....|....|....|....|....|....|
transcriptome ATTAAATAGGCCTGATATAGCTTTTCCACGGCTAAATAATATAAGATTTTGGTTATTGCCTCCATCTCTGTCATTATTATTAGGGTCAAGGTTACTAGGA
PS96_310R     ............G..............G................................A........A..............A...............
Ap199.3C      ...........................G........................................................................
Ap200.2E      ....................................................................................................
Ap225.2C      ....................................................................................................
Ap233.1E      ...........................G........................................................................

                      310       320       330       340       350       360       370       380       390       400
              ....|....|....|....|....|....|....|....|....|....|....|....|....|....|....|....|....|....|....|....|
transcriptome AGTGGGGCAGGGACCGGATGAACAGTATACCCTCCTTTGTCTAATTTAACGAATCATAGAGGCGGGTCGGTCGACATAGTTATTTTTTCTTTGCATATGG
PS96_310R     ..................................................T.................................................
Ap199.3C      ....................................................................................................
Ap200.2E      ..C.................................................................................................
Ap225.2C      ....................................................................................................
Ap233.1E      ....................................................................................................

                      410       420       430       440       450       460       470       480       490       500
              ....|....|....|....|....|....|....|....|....|....|....|....|....|....|....|....|....|....|....|....|
transcriptome CCGGAGCCAGAAGAATTTTAGCTTCAATTAATTTTATGGTCACTATTTATAATGGGCGGCCTAAAGCTCTAAGATATGATCGAGTAACTATATTTATTTG
PS96_310R     ........................................................................................C...........
Ap199.3C      ....................................................................................................
Ap200.2E      ....................................................................................................
Ap225.2C      ....................................................................................................
Ap233.1E      .T.......................M..........................................................................

                      510       520       530       540       550       560       570       580       590       600
              ....|....|....|....|....|....|....|....|....|....|....|....|....|....|....|....|....|....|....|....|
transcriptome GTCTATTGTAGTTACGGCTATTTTATTGATTTTAGCATTGCCCGTATTAGCGGGCGCTATTACAATGTTGTTATTTGATCGAAATTTTAGAACATCTTTC
PS96_310R     ....................................................................................................
Ap199.3C      ....................................................................................................
Ap200.2E      ....................................................................................................
Ap225.2C      ....................................................................................................
Ap233.1E      ....................................................................................................

                      610       620
              ....|....|....|....|....|.
transcriptome TTTGACCCCGCTGGCGGTGGCGATCC
PS96_310R     ..........................
Ap199.3C      ..........................
Ap200.2E      ..........................
Ap225.2C      ..........................
Ap233.1E      ..........................
```
