# Supplementary material for: Phylogenomics of Aplacophora (Mollusca, Aculifera) and a solenogaster without a foot
Source: Proc Biol Sci. 2019 May 8;286(1902):20190115. doi: 10.1098/rspb.2019.0115 (PMC6532501; doi:10.1098/rspb.2019.0115)
Supplement: Supplementary Tables [file rspb20190115supp9.doc]

**Supplementary Table 1.** Collection data for taxa from which new data were generated for this study.

| **Taxon** | **Collection locality** | **Collection method** | **Tissue used** | **Extraction method** | **Starved?** |
| --- | --- | --- | --- | --- | --- |
| *Alexandromenia crassa* | Møre og Romsdal, North of Sandsøyna, Norway (62° 16.70' N, 5° 27.25' E) | Epibenthic sled | Anterior half | TRIzol + RNEasy | Yes – 8 weeks |
| Amphimeniidae sp. | Wrigley Gulf, Amundsen Sea, Antarctica (73° 09.5316’ S, 129° 53.6975’ W) | Blake trawl | Piece of mid-body enriched for mantle tissue | TRIzol + RNEasy | No |
| *Apodomenia enigmatica* | Amundsen Sea, Antarctica (73° 17.7997’ S, 129° 11.5466’ W) | Blake trawl | Piece of mid-body enriched for mantle tissue | RNaqueous Micro | No |
| *Chaetoderma nitidulum* | Rogaland, Norway | Epibenthic sled | Entire animal | RNaqueous Micro | No |
| *Entonomenia tricarinata* | Ross Sea, Antarctica (76° 14.7157’ S, 174° 30.2472’ E) | Blake trawl | Piece of mid-body enriched for mantle tissue | TRIzol + RNEasy | No |
| *Epimenia babai* | Amakusa, Japan (collected by Akiko Okusu) | Hand collected | Piece of mid-body enriched for mantle tissue | TRIzol + RNEasy | No |
| *Falcidens caudatus* | North Carolina (35° 28.466' N, 74° 46.746' W) | Box corer | Entire animal | RNeasy Micro | No |
| *Falcidens sagittiferus* | Møre og Romsdal, Vartdalsfjorden North, Norway (62° 21.07' N, 6° 09.71' E) | Epibenthic sled | Entire animal | RNaqueous Micro | No |
| *Helluoherpia aegiri* | Hordaland, Hauglandsosen, Norway (60° 26.07' N, 5° 7.44' E) | Epibenthic sled | Entire animal | RNeasy Micro | Yes – 3 weeks |
| *Hypomenia sanjuanensis* | Friday Harbor, WA, USA (48° 32.6667' N, 122° 58.9667' W) | van Veen grab | Five entire animals | RNaqueous Micro | No |
| *Kruppomenia borealis* | Hordaland, Hauglandsosen, Norway (60° 26.07' N, 5° 7.44' E) | Epibenthic sled | Entire animal | RNaqueous Micro | Yes – 3 weeks |
| *Leptochiton rugatus* | Friday Harbor, WA, USA (48° 32.6667' N, 122° 58.9667' W) | van Veen grab | Entire animal | RNeasy Micro | No |
| *Macellomenia schanderi* | Friday Harbor, WA, USA (48° 32.6667' N, 122° 58.9667' W) | van Veen grab | Entire animal | RNaqueous Micro | Yes – 4 days |
| *Meiomenia swedmarki* | Friday Harbor, WA (48° 32.6667' N, 122° 58.9667' W) | van Veen grab | Entire animal | RNaqueous Micro | Yes – 4 days |
| *Micromenia fodiens* | Near Bergen, Norway | Epibenthic sled | Entire animal | RNaqueous Micro | Yes – 3 weeks |
| *Neomenia carinata* | Near Bergen, Norway | Epibenthic sled | Piece of mid-body enriched for mantle tissue | TRIzol + RNEasy | Yes – 8 weeks |
| *Phyllomenia* sp. | Ross Sea, Antarctica (75° 19.7781 S, 176° 59.1055 W) | Blake trawl | Section through mid-body | RNaqueous Micro | No |
| *Prochaetoderma californicum* | Oregon, USA (45° 49.784', 125° 9.996') | Multicorer | Entire animal | RNaqueous Micro | No |
| *Proneomenia sluiteri* | Northeastern Iceland (66° 17.72' N, 12° 21.76' W) | Agassiz trawl | Piece of mid-body enriched for mantle tissue | TRIzol + RNEasy | No |
| *Proneomenia custodiens* | Northeastern Iceland (66° 17.72' N, 12° 21.76' W) | Agassiz trawl | 8 unhatched juveniles | RNeasy Micro | N/A |
| *Simrothiella margaritacea* | Møre og Romsdal, North of Sandsøyna (62° 16.70' N, 5° 27.25' E) | Epibenthic sled | Posterior half | RNaqueous Micro | Yes – 8 weeks |
| *Spathoderma clenchi* | Iceland | Epibenthic sled | Entire animal | RNaqueous Micro | No |
| *Scutopus ventrolineatus* | Sognefjorden, Norway | Epibenthic sled | Entire animal | RNaqueous Micro | No |
| *Stylomenia sulcodoryata* | Rogaland, Norway | Epibenthic sled | Entire animal | RNaqueous Micro | No |
| *Tonicella lineata* | Cattle Point, San Juan Island, WA, USA | Hand collected | Mantle tissue | RNaqueous Micro | No |

**Supplementary Table 2.** Data analyzed in this study.

| **Taxon** | **Traditional systematics** | **Data** | **Reads** | **OGs / 525** | **OGs / 200** | **Accession, URL, etc.** |
| --- | --- | --- | --- | --- | --- | --- |
| *Alexandromenia crassa* | Solenogastres, Amphimeniidae | Illumina | 45,059,456 | 395 | 150 | SRR2052564 |
| Amphimeniidae sp. | Solenogastres, Amphimeniidae | Illumina | 49,911,520 | 458 | 184 | SRR6926328 |
| *Apodomenia enigmatica* | Solenogastres, Apodomeniidae  fam. nov. | Illumina | 61,008,716 | 392 | 140 | SRR6926325 |
| *Chaetoderma nitidulum* | Caudofoveata, Chaetodermatdiae | Illumina | 102,718,730 | 416 | 173 | SRR5341487 |
| *Crassostrea gigas* | Bivalvia | Genome | - | 460 | 177 | gigadb.org/Pacific_oyster |
| *Entonomenia tricarinata* | Solenogastres, Rhopalomeniidae | Illumina | 65,227,196 | 504 | 190 | SRR6926335 |
| *Epimenia babai* | Solenogastres, Epimeniidae | Illumina | 55,835,500 | 390 | 142 | SRR6926338 |
| *Falcidens caudatus* | Caudofoveata, Chaetodermatdiae | Illumina | 49,904,154 | 382 | 136 | SRR6926337 |
| *Falcidens sagittiferus* | Caudofoveata, Chaetodermatdiae | Illumina | 106,189,960 | 483 | 189 | SRR6926339 |
| *Helluoherpia aegiri* | Solenogastres, Dondersiidae | Illumina | 63,723,082 | 373 | 140 | SRR6926336 |
| *Hypomenia sanjuanensis* | Solenogastres, Pruvotinidae | Illumina | 42,284,504 | 359 | 129 | SRR6926334 |
| *Kruppomenia borealis* | Solenogastres, Simrothiellidae | Illumina | 50,526,030 | 248 | 89 | SRR6926323 |
| *Leptochiton rugatus* | Polyplacophora | Illumina | 49,670,054 | 423 | 160 | SRR1611558 |
| *Lottia gigantea* | Gastropoda | Genome | - | 501 | 191 | JGI filtered models v. 1.0 |
| *Macellomenia schanderi* | Solenogastres, Macellomeniidae | Illumina | 49,904,154 | 500 | 186 | SRR2057023 |
| *Meiomenia swedmarki* | Solenogastres, Meiomeniidae | Illumina | 41,521,194 | 339 | 131 | SRR6926324 |
| *Micromenia fodiens* | Solenogastres, Dondersiidae | Illumina | 60,286,842 | 425 | 161 | SRR6926322 |
| *Neomenia* *carinata* | Solenogastres, Neomeniidae | Illumina | 36,612,396 | 281 | 96 | SRR2057026 |
| *Neomenia megatrapezata* | Solenogastres, Neomeniidae | Illumina | 58,583,176 | 412 | 154 | datadryad.org/handle/10255/dryad.34644 |
| Pholidoskepia sp. | Solenogastres | Illumina | 10,131,129 | 187 | 65 | SRR1505105 |
| *Phyllomenia* sp. | Solenogastres, Phyllomeniidae | Illumina | 36,837,056 | 222 | 85 | SRR6926329 |
| *Prochaetoderma californicum* | Caudofoveata, Prochaetodermatidae | Illumina | 90,561,352 | 395 | 156 | SRR6926326 |
| *Proneomenia* *custodiens* | Solenogastres, Proneomeniidae | Illumina | 43,590,954 | 463 | 170 | SRR1611561 |
| *Proneomenia sluiteri* | Solenogastres, Proneomeniidae | Illumina | 46,763,538 | 269 | 92 | SRR6926327 |
| Pruvotinidae sp. Greenland | Solenogastres, Pruvotinidae | Illumina | 70,456,224 | 377 | 144 | datadryad.org/handle/10255/dryad.34644 |
| *Rhyssoplax olivaceus* | Polyplacophora | Illumina | 23,189,291 | 436 | 175 | SRR618506 |
| *Scutopus ventrolineatus* | Caudofoveata, Limifossoridae | Illumina | 23,189,291 | 346 | 150 | SRR6926333 |
| *Simrothiella margaritacea* | Solenogastres, Simrothiellidae | Illumina | 34,777,076 | 307 | 110 | SRR6926332 |
| *Spathoderma clenchi* | Caudofoveata, Prochaetodermatidae | Illumina | 52,062,402 | 220 | 87 | SRR8258011 |
| *Stylomenia sulcodoryata* | Solenogastres, Dondersiidae | Illumina | 111,501,252 | 504 | 191 | SRR6926330 |
| *Tonicella lineata* | Polyplacophora | Illumina | 105,180,728 | 480 | 185 | SRR6926331 |
| *Wirenia argentea* | Solenogastres, Gymnomeniidae | Illumina | 100,913,778 | 459 | 173 | SRR5110527 |

Transcriptomes generated for this study are indicated in blue text.

**Supplementary Table 3.** Characteristics of data matrices analyzed.

| **Matrix** | **Taxa** | **Number of genes** | **Number of positions** | **Percent missing data** |
| --- | --- | --- | --- | --- |
| Complete | 32 | 525 | 75,914 | 30.43% |
| Complete (no *F. caudatus*) | 31 | 525 | 75,914 | 30.36% |
| Reduced LB | 32 | 200 | 30,185 | 30.70% |

**Supplementary Table 4.** Characteristics of all OGs analyzed.

| **OG** | **Taxa** | **RCFV** | **LB Score** | **Avg. PD** | **Slope** | **R^2^** | **Avg. BS** |
| --- | --- | --- | --- | --- | --- | --- | --- |
| 00058 | 23 | 0.003304 | 28.562224 | 0.284721 | 0.284756 | 0.695183 | 32.10 |
| 00059 | 21 | 0.007852 | 14.346654 | 1.447346 | 0.268336 | 0.855598 | 49.33 |
| 00060 | 24 | 0.003892 | 51.816514 | 0.633531 | 0.188748 | 0.779887 | 49.48 |
| 00073 | 22 | 0.007705 | 29.713377 | 0.827378 | 0.186134 | 0.573830 | 39.32 |
| 00108 | 26 | 0.005785 | 57.299375 | 1.120835 | 0.146898 | 0.725739 | 47.65 |
| 00137 | 24 | 0.005358 | 26.070417 | 0.854899 | 0.267254 | 0.911086 | 66.48 |
| 00147 | 23 | 0.004735 | 15.951662 | 0.662488 | 0.361244 | 0.766270 | 72.50 |
| 00155 | 30 | 0.006157 | 33.274214 | 1.113808 | 0.218643 | 0.790619 | 64.48 |
| 00158 | 28 | 0.006082 | 18.828423 | 0.726107 | 0.231943 | 0.559560 | 43.28 |
| 00180 | 25 | 0.003908 | 50.092962 | 0.412563 | 0.117069 | 0.420458 | 41.82 |
| 00192 | 22 | 0.006464 | 26.657716 | 0.640595 | 0.262411 | 0.494675 | 44.05 |
| 00206 | 28 | 0.004996 | 18.633886 | 1.149131 | 0.160099 | 0.472063 | 57.64 |
| 00212 | 20 | 0.006440 | 41.611888 | 1.260098 | 0.114223 | 0.400536 | 41.76 |
| 00215 | 23 | 0.006848 | 22.557338 | 1.186005 | 0.261099 | 0.849343 | 69.60 |
| 00223 | 24 | 0.003317 | 19.508807 | 0.589537 | 0.296587 | 0.775455 | 43.24 |
| 00237 | 25 | 0.006352 | 16.094030 | 1.162140 | 0.253090 | 0.698682 | 69.00 |
| 00239 | 28 | 0.006757 | 48.983376 | 1.413074 | 0.151467 | 0.779108 | 49.04 |
| 00255 | 25 | 0.005168 | 29.250972 | 0.630366 | 0.297310 | 0.737465 | 59.59 |
| 00275 | 25 | 0.004072 | 16.695906 | 0.444078 | 0.319267 | 0.596931 | 56.82 |
| 00280 | 23 | 0.005048 | 15.649740 | 0.845611 | 0.322941 | 0.754435 | 58.15 |
| 00288 | 21 | 0.005181 | 52.436141 | 0.682930 | 0.236847 | 0.852531 | 35.61 |
| 00321 | 26 | 0.004469 | 159.967368 | 1.941688 | 0.022737 | 0.726017 | 28.04 |
| 00350 | 21 | 0.009505 | 10.249781 | 0.817273 | 0.259148 | 0.744533 | 37.94 |
| 00362 | 24 | 0.005933 | 40.315153 | 0.802959 | 0.311595 | 0.896144 | 44.00 |
| 00376 | 26 | 0.006919 | 24.624295 | 1.262260 | 0.200326 | 0.665792 | 48.52 |
| 00391 | 25 | 0.006512 | 40.528508 | 0.825274 | 0.237952 | 0.773052 | 45.14 |
| 00406 | 22 | 0.004655 | 20.429418 | 0.398540 | 0.508293 | 0.894912 | 48.05 |
| 00407 | 24 | 0.005796 | 31.314357 | 0.439841 | 0.436661 | 0.843992 | 63.67 |
| 00435 | 23 | 0.004935 | 35.639983 | 0.911504 | 0.278779 | 0.879225 | 59.25 |
| 00443 | 23 | 0.007330 | 28.188396 | 1.252404 | 0.216712 | 0.720366 | 57.65 |
| 00450 | 26 | 0.006985 | 39.496400 | 0.942723 | 0.208157 | 0.680964 | 59.78 |
| 00461 | 24 | 0.003975 | 129.456265 | 1.159085 | 0.064765 | 0.726943 | 53.90 |
| 00463 | 20 | 0.004860 | 17.076688 | 0.663342 | 0.440952 | 0.942554 | 73.65 |
| 00475 | 22 | 0.006864 | 15.496634 | 1.201582 | 0.255456 | 0.622631 | 39.42 |
| 00480 | 23 | 0.005457 | 23.984460 | 0.793652 | 0.307462 | 0.788499 | 60.95 |
| 00481 | 24 | 0.006225 | 37.258153 | 0.786744 | 0.275056 | 0.901790 | 47.24 |
| 00491 | 21 | 0.005295 | 12.576637 | 0.764170 | 0.330695 | 0.762820 | 69.61 |
| 00495 | 20 | 0.006440 | 19.974533 | 1.426228 | 0.199629 | 0.508069 | 69.53 |
| 00500 | 27 | 0.004941 | 28.474971 | 0.975640 | 0.265297 | 0.795349 | 52.25 |
| 00519 | 27 | 0.005615 | 65.011946 | 0.641180 | 0.213026 | 0.807717 | 41.79 |
| 00540 | 20 | 0.005250 | 23.670967 | 1.019257 | 0.241098 | 0.750757 | 71.94 |
| 00558 | 22 | 0.005432 | 11.849303 | 1.005291 | 0.280617 | 0.667129 | 70.84 |
| 00581 | 28 | 0.008871 | 16.936187 | 1.271421 | 0.223570 | 0.672149 | 51.28 |
| 00584 | 23 | 0.006165 | 20.408441 | 0.938546 | 0.299701 | 0.729370 | 59.10 |
| 00627 | 20 | 0.007675 | 25.842776 | 0.356492 | 0.465796 | 0.842485 | 47.59 |
| 00667 | 26 | 0.004958 | 53.289462 | 0.950626 | 0.163477 | 0.716758 | 39.22 |
| 00669 | 25 | 0.006096 | 20.486335 | 1.075409 | 0.223957 | 0.700611 | 53.14 |
| 00684 | 24 | 0.006288 | 33.221771 | 0.540875 | 0.275238 | 0.827802 | 53.43 |
| 00693 | 22 | 0.005723 | 15.969558 | 1.212888 | 0.250473 | 0.822052 | 49.05 |
| 00696 | 25 | 0.005676 | 27.148595 | 0.507318 | 0.357783 | 0.645581 | 61.59 |
| 00706 | 20 | 0.004760 | 39.981001 | 0.382785 | 0.326232 | 0.861298 | 39.71 |
| 00719 | 23 | 0.005470 | 51.019505 | 0.636223 | 0.237769 | 0.761693 | 39.85 |
| 00721 | 26 | 0.003638 | 19.877808 | 0.418033 | 0.393452 | 0.897406 | 52.13 |
| 00817 | 20 | 0.006975 | 12.509372 | 0.977182 | 0.297345 | 0.848519 | 63.29 |
| 00823 | 29 | 0.006800 | 24.746299 | 0.803778 | 0.258276 | 0.669361 | 55.46 |
| 00826 | 25 | 0.002840 | 52.911180 | 0.152771 | 0.506118 | 0.928722 | 34.95 |
| 00828 | 26 | 0.006335 | 33.905642 | 1.074494 | 0.192528 | 0.687050 | 61.43 |
| 00832 | 28 | 0.006236 | 18.912817 | 0.867157 | 0.334606 | 0.870982 | 64.72 |
| 00834 | 27 | 0.006370 | 19.124336 | 0.872543 | 0.314591 | 0.823794 | 60.17 |
| 00837 | 23 | 0.005552 | 10.274979 | 0.912952 | 0.291075 | 0.709009 | 73.75 |
| 00844 | 22 | 0.003873 | 44.259294 | 0.194105 | 0.441187 | 0.887107 | 30.68 |
| 00848 | 23 | 0.006926 | 17.595057 | 1.251632 | 0.206147 | 0.626622 | 43.05 |
| 00849 | 22 | 0.005805 | 17.519692 | 0.960907 | 0.253113 | 0.787012 | 48.37 |
| 00856 | 25 | 0.005764 | 15.808016 | 0.882531 | 0.273312 | 0.563506 | 51.27 |
| 00857 | 24 | 0.005658 | 44.818667 | 1.156646 | 0.159371 | 0.604502 | 56.48 |
| 00867 | 24 | 0.011033 | 27.965387 | 1.466772 | 0.211957 | 0.784338 | 25.38 |
| 00883 | 25 | 0.003572 | 83.749484 | 0.317965 | 0.310414 | 0.875353 | 45.23 |
| 00911 | 20 | 0.006135 | 54.173074 | 0.930551 | 0.188780 | 0.732986 | 33.94 |
| 00916 | 25 | 0.005968 | 30.376118 | 1.473962 | 0.166837 | 0.692298 | 52.91 |
| 00919 | 26 | 0.004508 | 19.990196 | 0.645667 | 0.338347 | 0.758745 | 63.61 |
| 00920 | 28 | 0.003954 | 28.212347 | 0.369008 | 0.379348 | 0.882454 | 51.40 |
| 00922 | 28 | 0.006564 | 18.615969 | 1.229305 | 0.260032 | 0.730602 | 67.60 |
| 00942 | 23 | 0.005591 | 35.470746 | 0.764888 | 0.307462 | 0.877052 | 55.90 |
| 00946 | 26 | 0.006792 | 38.698818 | 1.064969 | 0.195272 | 0.670359 | 56.30 |
| 00947 | 25 | 0.005308 | 27.590999 | 0.408161 | 0.320365 | 0.804308 | 44.50 |
| 00958 | 21 | 0.004762 | 32.023456 | 0.462160 | 0.411081 | 0.901443 | 55.83 |
| 00983 | 24 | 0.005708 | 27.173133 | 0.576676 | 0.369012 | 0.854574 | 45.33 |
| 01000 | 23 | 0.006970 | 40.535855 | 0.841166 | 0.237974 | 0.768042 | 36.80 |
| 01008 | 24 | 0.005346 | 18.148529 | 0.591461 | 0.380452 | 0.727155 | 59.76 |
| 01012 | 23 | 0.003761 | 127.376684 | 0.373930 | 0.212403 | 0.903959 | 34.00 |
| 01025 | 21 | 0.004500 | 75.537851 | 1.054406 | 0.130396 | 0.764766 | 45.67 |
| 01031 | 21 | 0.006705 | 11.080084 | 1.084392 | 0.297779 | 0.659153 | 71.50 |
| 01035 | 21 | 0.007886 | 25.714496 | 1.255032 | 0.204485 | 0.753818 | 55.44 |
| 01047 | 26 | 0.006438 | 23.093818 | 0.848954 | 0.339332 | 0.848635 | 55.09 |
| 01066 | 27 | 0.003956 | 69.639167 | 0.529796 | 0.201464 | 0.819827 | 48.67 |
| 01073 | 24 | 0.004738 | 23.952950 | 0.688851 | 0.349356 | 0.895821 | 63.86 |
| 01087 | 24 | 0.004600 | 24.735646 | 0.344441 | 0.474644 | 0.883415 | 40.48 |
| 01092 | 23 | 0.006091 | 21.163113 | 0.980165 | 0.302268 | 0.659088 | 62.90 |
| 01096 | 24 | 0.005013 | 28.108754 | 0.644704 | 0.281104 | 0.766248 | 57.76 |
| 01097 | 26 | 0.005769 | 35.708897 | 0.484673 | 0.356466 | 0.845055 | 50.52 |
| 01107 | 25 | 0.004932 | 34.735402 | 0.532271 | 0.317906 | 0.817642 | 46.95 |
| 01135 | 20 | 0.005430 | 29.012634 | 0.435951 | 0.239886 | 0.526584 | 38.82 |
| 01137 | 28 | 0.005154 | 52.721719 | 0.620413 | 0.231328 | 0.798300 | 40.20 |
| 01140 | 24 | 0.005996 | 19.577499 | 0.950991 | 0.220188 | 0.493580 | 69.10 |
| 01145 | 23 | 0.008517 | 13.449659 | 0.930977 | 0.358526 | 0.860904 | 48.80 |
| 01160 | 30 | 0.005967 | 20.953970 | 0.690930 | 0.319472 | 0.826618 | 54.63 |
| 01174 | 26 | 0.003019 | 33.440329 | 0.289302 | 0.337271 | 0.830896 | 47.00 |
| 01177 | 20 | 0.006010 | 42.483720 | 0.922792 | 0.061055 | 0.097755 | 49.00 |
| 01179 | 25 | 0.006612 | 17.871905 | 0.767392 | 0.318255 | 0.750663 | 56.64 |
| 01180 | 27 | 0.005844 | 52.735047 | 0.722583 | 0.228427 | 0.782418 | 52.88 |
| 01198 | 27 | 0.006415 | 42.826877 | 1.027410 | 0.164876 | 0.695216 | 41.46 |
| 01202 | 25 | 0.006824 | 13.581801 | 0.844354 | 0.358035 | 0.837547 | 64.86 |
| 01206 | 25 | 0.006352 | 15.419749 | 0.993155 | 0.272778 | 0.600984 | 68.77 |
| 01219 | 22 | 0.004764 | 25.162372 | 0.508620 | 0.289157 | 0.633123 | 44.32 |
| 01233 | 25 | 0.005244 | 21.545846 | 0.955098 | 0.330757 | 0.888008 | 51.05 |
| 01240 | 28 | 0.005711 | 21.765394 | 0.708654 | 0.361205 | 0.769777 | 66.44 |
| 01255 | 23 | 0.004783 | 32.356859 | 0.218247 | 0.548755 | 0.875045 | 36.65 |
| 01258 | 27 | 0.007507 | 56.157396 | 1.136669 | 0.113306 | 0.534710 | 47.88 |
| 01265 | 23 | 0.006417 | 30.499437 | 0.792998 | 0.269827 | 0.790815 | 47.05 |
| 01267 | 24 | 0.004167 | 22.821031 | 0.590258 | 0.343294 | 0.908117 | 52.90 |
| 01278 | 28 | 0.003907 | 30.254609 | 0.397858 | 0.385621 | 0.883058 | 57.60 |
| 01279 | 21 | 0.006190 | 22.964115 | 0.849119 | 0.316439 | 0.796340 | 66.39 |
| 01320 | 25 | 0.006396 | 63.244558 | 1.483051 | 0.086058 | 0.551048 | 45.00 |
| 01331 | 22 | 0.004491 | 84.911268 | 0.586920 | 0.216557 | 0.913260 | 40.95 |
| 01334 | 22 | 0.005177 | 11.279078 | 0.732687 | 0.333735 | 0.749681 | 66.89 |
| 01336 | 28 | 0.006332 | 17.146370 | 0.784289 | 0.351530 | 0.729469 | 60.72 |
| 01339 | 22 | 0.005814 | 21.921646 | 0.866746 | 0.287422 | 0.777827 | 57.89 |
| 01341 | 21 | 0.004610 | 44.878962 | 0.285823 | 0.326153 | 0.813333 | 36.56 |
| 01343 | 20 | 0.005895 | 36.992683 | 0.928686 | 0.091553 | 0.175610 | 41.88 |
| 01360 | 25 | 0.003444 | 59.131279 | 0.336835 | 0.320256 | 0.832386 | 59.41 |
| 01375 | 23 | 0.004952 | 80.714331 | 0.783490 | 0.143499 | 0.719230 | 49.85 |
| 01376 | 24 | 0.006392 | 24.636108 | 1.085408 | 0.220254 | 0.761412 | 48.90 |
| 01393 | 22 | 0.006214 | 41.323313 | 1.163824 | 0.120595 | 0.469277 | 30.84 |
| 01409 | 26 | 0.004158 | 37.673225 | 0.550549 | 0.341998 | 0.807111 | 69.35 |
| 01413 | 20 | 0.005540 | 21.777499 | 0.472019 | 0.295447 | 0.659511 | 53.88 |
| 01422 | 27 | 0.002578 | 20.450238 | 0.316905 | 0.313778 | 0.673596 | 73.13 |
| 01425 | 26 | 0.005231 | 17.845958 | 0.721100 | 0.345942 | 0.787774 | 62.57 |
| 01431 | 22 | 0.004282 | 65.378691 | 0.606507 | 0.207435 | 0.811686 | 43.95 |
| 01435 | 28 | 0.004075 | 72.706951 | 0.640957 | 0.180214 | 0.742793 | 46.68 |
| 01440 | 21 | 0.006871 | 33.014748 | 0.692008 | 0.306397 | 0.855544 | 38.61 |
| 01467 | 25 | 0.007452 | 15.901454 | 1.159965 | 0.245393 | 0.777015 | 52.64 |
| 01470 | 27 | 0.004652 | 61.805526 | 0.555241 | 0.268083 | 0.836667 | 43.25 |
| 01477 | 25 | 0.004056 | 50.142962 | 0.407796 | 0.305039 | 0.860812 | 51.82 |
| 01493 | 21 | 0.007390 | 43.806493 | 0.969449 | 0.238264 | 0.836056 | 46.67 |
| 01495 | 21 | 0.006924 | 14.082113 | 1.095162 | 0.298365 | 0.858424 | 72.28 |
| 01560 | 23 | 0.005283 | 73.974704 | 0.943776 | 0.106723 | 0.563779 | 40.10 |
| 01569 | 20 | 0.005250 | 40.118914 | 0.792913 | 0.222170 | 0.698221 | 49.59 |
| 01585 | 27 | 0.003474 | 69.074789 | 0.687357 | 0.157697 | 0.794109 | 51.58 |
| 01586 | 20 | 0.005270 | 120.193935 | 1.035177 | 0.029883 | 0.264613 | 53.18 |
| 01591 | 21 | 0.004643 | 88.812708 | 0.746226 | 0.196688 | 0.902436 | 39.72 |
| 01594 | 27 | 0.003252 | 23.253816 | 0.408205 | 0.351220 | 0.746347 | 54.88 |
| 01596 | 29 | 0.005145 | 57.893172 | 0.854520 | 0.162185 | 0.582184 | 52.08 |
| 01598 | 23 | 0.007696 | 44.343983 | 1.080540 | 0.174910 | 0.691931 | 51.50 |
| 01607 | 25 | 0.005292 | 16.845946 | 0.867877 | 0.278348 | 0.738034 | 67.00 |
| 01609 | 30 | 0.005563 | 72.641475 | 0.848606 | 0.106705 | 0.541003 | 49.74 |
| 01619 | 23 | 0.006230 | 14.355554 | 0.893613 | 0.247667 | 0.499416 | 61.40 |
| 01626 | 24 | 0.006017 | 26.463422 | 1.018444 | 0.264668 | 0.796634 | 57.90 |
| 01628 | 25 | 0.008320 | 29.889376 | 1.471641 | 0.134815 | 0.443083 | 45.23 |
| 01633 | 28 | 0.005396 | 20.702257 | 0.749727 | 0.298649 | 0.850933 | 52.88 |
| 01669 | 25 | 0.004048 | 20.695712 | 0.470093 | 0.236669 | 0.378987 | 48.23 |
| 01677 | 20 | 0.007910 | 39.455746 | 1.042746 | 0.189198 | 0.686024 | 30.35 |
| 01688 | 24 | 0.005192 | 9.416342 | 1.106247 | 0.271687 | 0.644925 | 64.00 |
| 01701 | 26 | 0.005919 | 12.452448 | 1.131768 | 0.260548 | 0.782217 | 71.83 |
| 01705 | 22 | 0.007050 | 18.533711 | 1.235916 | 0.252953 | 0.701192 | 52.47 |
| 01709 | 24 | 0.005029 | 18.873004 | 0.530339 | 0.344897 | 0.598193 | 50.67 |
| 01735 | 22 | 0.003764 | 16.345908 | 0.520390 | 0.336282 | 0.775215 | 54.74 |
| 01737 | 22 | 0.006191 | 41.365413 | 0.884713 | 0.233758 | 0.843404 | 43.84 |
| 01763 | 24 | 0.006046 | 59.299504 | 0.809965 | 0.208186 | 0.781173 | 43.10 |
| 01788 | 25 | 0.005776 | 18.250360 | 0.803483 | 0.318850 | 0.854977 | 52.05 |
| 01822 | 21 | 0.006257 | 16.713964 | 0.915568 | 0.319363 | 0.777340 | 54.61 |
| 01851 | 24 | 0.007279 | 14.261234 | 1.093775 | 0.342026 | 0.826832 | 46.86 |
| 01860 | 21 | 0.004710 | 17.853360 | 1.206139 | 0.202930 | 0.697102 | 60.94 |
| 01862 | 28 | 0.005614 | 58.225299 | 1.045135 | 0.133658 | 0.729209 | 43.56 |
| 01863 | 28 | 0.005546 | 48.117390 | 0.741558 | 0.224479 | 0.835791 | 39.20 |
| 01890 | 30 | 0.006313 | 20.518775 | 1.103034 | 0.214916 | 0.648206 | 64.96 |
| 01893 | 23 | 0.004822 | 75.726705 | 0.325425 | 0.367040 | 0.928166 | 40.40 |
| 01906 | 23 | 0.004852 | 27.956459 | 0.777904 | 0.236498 | 0.698346 | 37.70 |
| 01907 | 25 | 0.005828 | 54.733489 | 0.566992 | 0.217098 | 0.857569 | 28.18 |
| 01919 | 21 | 0.004233 | 25.155083 | 0.719243 | 0.229185 | 0.644544 | 61.00 |
| 01925 | 23 | 0.006052 | 180.956472 | 1.138370 | 0.028222 | 0.535804 | 44.80 |
| 01934 | 24 | 0.007888 | 18.365663 | 1.286345 | 0.210731 | 0.605524 | 65.14 |
| 01950 | 26 | 0.006038 | 40.283852 | 1.141208 | 0.132191 | 0.516285 | 36.09 |
| 01962 | 22 | 0.006245 | 25.624728 | 1.070281 | 0.209597 | 0.438993 | 55.63 |
| 01989 | 22 | 0.006559 | 19.998461 | 1.273880 | 0.234381 | 0.768589 | 56.37 |
| 02009 | 24 | 0.005017 | 104.964300 | 1.866268 | 0.036307 | 0.530066 | 26.95 |
| 02011 | 20 | 0.006620 | 44.415924 | 1.251123 | 0.149503 | 0.650901 | 47.41 |
| 02020 | 20 | 0.005470 | 57.041518 | 0.528527 | 0.303255 | 0.900476 | 41.71 |
| 02027 | 20 | 0.006945 | 15.633918 | 0.652665 | 0.308189 | 0.722801 | 42.29 |
| 02040 | 21 | 0.007567 | 14.633142 | 0.976467 | 0.254884 | 0.693477 | 58.44 |
| 02068 | 25 | 0.005092 | 17.881664 | 0.696824 | 0.302304 | 0.540238 | 59.14 |
| 02073 | 22 | 0.007827 | 32.089559 | 0.912728 | 0.164887 | 0.372212 | 25.74 |
| 02092 | 24 | 0.007742 | 10.366649 | 1.498895 | 0.235474 | 0.785945 | 57.10 |
| 02097 | 22 | 0.005168 | 31.559155 | 0.601785 | 0.361723 | 0.882156 | 51.68 |
| 02102 | 25 | 0.006148 | 37.332502 | 0.994109 | 0.179809 | 0.656206 | 55.77 |
| 02125 | 22 | 0.008582 | 13.194519 | 1.375524 | 0.245207 | 0.707504 | 48.21 |
| 02133 | 25 | 0.003960 | 161.002109 | 0.829165 | 0.081966 | 0.883442 | 39.27 |
| 02146 | 27 | 0.003885 | 68.068098 | 0.396662 | 0.323841 | 0.952047 | 50.50 |
| 02150 | 24 | 0.008238 | 30.745011 | 0.814318 | 0.295510 | 0.742331 | 32.90 |
| 02152 | 23 | 0.003896 | 21.449269 | 0.240230 | 0.512655 | 0.778151 | 48.30 |
| 02182 | 23 | 0.005304 | 9.979497 | 1.218574 | 0.267519 | 0.633069 | 70.00 |
| 02188 | 24 | 0.007192 | 19.787861 | 1.437582 | 0.251292 | 0.849367 | 57.48 |
| 02196 | 23 | 0.005817 | 24.708299 | 0.781489 | 0.291856 | 0.811630 | 50.65 |
| 02219 | 25 | 0.003368 | 60.076694 | 0.375659 | 0.307122 | 0.822243 | 55.50 |
| 02233 | 22 | 0.004509 | 41.378347 | 0.913945 | 0.137862 | 0.452631 | 54.11 |
| 02245 | 26 | 0.005558 | 120.336858 | 0.799735 | 0.064176 | 0.413798 | 40.04 |
| 02250 | 26 | 0.006319 | 29.064991 | 1.001127 | 0.149820 | 0.350984 | 46.30 |
| 02251 | 29 | 0.007614 | 21.899167 | 1.294415 | 0.213450 | 0.767669 | 52.00 |
| 02255 | 21 | 0.006033 | 30.960460 | 0.949181 | 0.169381 | 0.467149 | 35.89 |
| 02261 | 26 | 0.007304 | 29.387887 | 1.049575 | 0.242641 | 0.755483 | 50.39 |
| 02270 | 21 | 0.007429 | 19.959075 | 1.498615 | 0.190310 | 0.596940 | 46.39 |
| 02282 | 23 | 0.003535 | 22.188348 | 0.285997 | 0.364730 | 0.828475 | 68.90 |
| 02290 | 21 | 0.007505 | 16.483168 | 0.662092 | 0.299941 | 0.557867 | 47.17 |
| 02299 | 23 | 0.005926 | 13.506824 | 1.183432 | 0.271962 | 0.805462 | 62.90 |
| 02309 | 23 | 0.006357 | 22.734699 | 0.452916 | 0.414345 | 0.794694 | 31.60 |
| 02324 | 25 | 0.005924 | 17.597696 | 1.054249 | 0.237843 | 0.599261 | 43.27 |
| 02325 | 21 | 0.008771 | 46.179747 | 3.799392 | 0.053586 | 0.799043 | 38.17 |
| 02331 | 25 | 0.006144 | 61.266047 | 1.266824 | 0.112004 | 0.708749 | 39.64 |
| 02340 | 24 | 0.007667 | 59.454250 | 1.585266 | 0.127603 | 0.869756 | 39.38 |
| 02341 | 23 | 0.005565 | 21.095591 | 1.017425 | 0.252804 | 0.806069 | 61.40 |
| 02342 | 25 | 0.005436 | 16.246413 | 0.876555 | 0.223431 | 0.554523 | 44.36 |
| 02344 | 27 | 0.004930 | 29.781226 | 0.543937 | 0.412890 | 0.908845 | 55.46 |
| 02378 | 20 | 0.008305 | 11.380027 | 1.202656 | 0.255702 | 0.622472 | 48.24 |
| 02400 | 22 | 0.007473 | 26.190260 | 0.786286 | 0.344911 | 0.860976 | 39.11 |
| 02427 | 21 | 0.005362 | 93.629278 | 0.857362 | 0.096439 | 0.678982 | 45.33 |
| 02430 | 25 | 0.004448 | 106.221372 | 0.666737 | 0.176902 | 0.906776 | 33.45 |
| 02432 | 20 | 0.008560 | 57.610555 | 0.964351 | 0.132707 | 0.558819 | 34.29 |
| 02434 | 24 | 0.004113 | 93.269902 | 0.819828 | 0.078409 | 0.604522 | 48.38 |
| 02453 | 27 | 0.004452 | 26.382241 | 0.890393 | 0.292106 | 0.855590 | 66.63 |
| 02457 | 23 | 0.003591 | 13.056279 | 0.513994 | 0.242778 | 0.627330 | 67.55 |
| 02458 | 21 | 0.006571 | 18.004922 | 1.024623 | 0.306034 | 0.767974 | 77.50 |
| 02468 | 27 | 0.007393 | 34.613156 | 1.165615 | 0.184298 | 0.550753 | 49.79 |
| 02470 | 25 | 0.004172 | 108.908173 | 0.666217 | 0.127655 | 0.820535 | 42.55 |
| 02479 | 22 | 0.004427 | 29.433520 | 0.433347 | 0.310617 | 0.673812 | 68.63 |
| 02481 | 25 | 0.007376 | 124.318272 | 1.087109 | 0.090803 | 0.846059 | 26.36 |
| 02485 | 26 | 0.005515 | 38.740079 | 0.965167 | 0.244520 | 0.799378 | 61.65 |
| 02486 | 20 | 0.007265 | 17.724483 | 1.074044 | 0.314622 | 0.885001 | 54.29 |
| 02490 | 21 | 0.008229 | 55.309030 | 0.729781 | 0.212631 | 0.728227 | 36.61 |
| 02492 | 23 | 0.007191 | 34.902628 | 1.213834 | 0.155854 | 0.617439 | 24.85 |
| 02500 | 29 | 0.005814 | 13.964491 | 0.799331 | 0.294301 | 0.732583 | 68.19 |
| 02507 | 24 | 0.005950 | 14.506420 | 0.804383 | 0.321980 | 0.781190 | 43.24 |
| 02514 | 22 | 0.006882 | 20.824187 | 1.085684 | 0.260732 | 0.604941 | 70.05 |
| 02527 | 28 | 0.003946 | 36.881112 | 0.374474 | 0.263113 | 0.615984 | 64.64 |
| 02529 | 20 | 0.009670 | 14.389493 | 1.229078 | 0.233449 | 0.581924 | 61.24 |
| 02534 | 25 | 0.005796 | 22.021059 | 0.830537 | 0.306887 | 0.753139 | 57.86 |
| 02556 | 26 | 0.005573 | 31.076783 | 0.827100 | 0.263167 | 0.794017 | 63.74 |
| 02560 | 26 | 0.006365 | 56.830605 | 0.699709 | 0.246694 | 0.847056 | 46.13 |
| 02565 | 23 | 0.004370 | 69.986061 | 0.407338 | 0.184389 | 0.646842 | 47.30 |
| 02567 | 21 | 0.006548 | 21.700089 | 0.830029 | 0.300083 | 0.776438 | 64.67 |
| 02613 | 24 | 0.004204 | 26.940763 | 0.763815 | 0.250722 | 0.654219 | 52.95 |
| 02633 | 20 | 0.006575 | 22.086308 | 1.071596 | 0.232442 | 0.678687 | 39.59 |
| 02637 | 27 | 0.004959 | 23.134841 | 0.660355 | 0.273831 | 0.709712 | 51.29 |
| 02639 | 24 | 0.007963 | 10.733047 | 0.740644 | 0.360102 | 0.623775 | 57.48 |
| 02640 | 21 | 0.006081 | 27.811659 | 1.300496 | 0.226895 | 0.788864 | 61.83 |
| 02666 | 21 | 0.010405 | 21.971309 | 1.502499 | 0.168096 | 0.618995 | 39.06 |
| 02667 | 23 | 0.006774 | 31.717671 | 1.150356 | 0.205045 | 0.727892 | 58.85 |
| 02673 | 27 | 0.004137 | 14.442090 | 0.576478 | 0.374073 | 0.788859 | 65.38 |
| 02676 | 21 | 0.005514 | 15.962898 | 0.992252 | 0.193203 | 0.387616 | 61.17 |
| 02683 | 22 | 0.008582 | 54.928264 | 0.958611 | 0.079440 | 0.180955 | 44.63 |
| 02684 | 24 | 0.005458 | 86.385137 | 0.591257 | 0.209184 | 0.810517 | 44.57 |
| 02688 | 21 | 0.005776 | 16.270729 | 0.789250 | 0.303565 | 0.777455 | 42.61 |
| 02693 | 24 | 0.006017 | 34.215775 | 0.780743 | 0.318349 | 0.846712 | 68.29 |
| 02697 | 25 | 0.006592 | 13.506791 | 1.403138 | 0.251976 | 0.581808 | 62.68 |
| 02700 | 22 | 0.005591 | 18.971164 | 0.789616 | 0.285077 | 0.800339 | 56.37 |
| 02713 | 25 | 0.006740 | 20.604188 | 1.175387 | 0.224044 | 0.722960 | 47.23 |
| 02733 | 23 | 0.004387 | 30.010760 | 0.292583 | 0.459728 | 0.854903 | 58.85 |
| 02753 | 20 | 0.007730 | 10.205366 | 1.197324 | 0.249023 | 0.723223 | 54.18 |
| 02758 | 26 | 0.006585 | 57.148220 | 0.778607 | 0.237506 | 0.829564 | 48.52 |
| 02771 | 23 | 0.006248 | 19.630245 | 1.307404 | 0.220536 | 0.661473 | 62.80 |
| 02800 | 25 | 0.004384 | 26.309355 | 0.434265 | 0.392405 | 0.872881 | 38.73 |
| 02893 | 21 | 0.005119 | 28.291025 | 0.639266 | 0.346033 | 0.930025 | 55.72 |
| 02898 | 22 | 0.006464 | 12.813677 | 0.696333 | 0.250724 | 0.700882 | 57.32 |
| 02907 | 26 | 0.005927 | 23.788580 | 1.098706 | 0.217288 | 0.701020 | 64.83 |
| 02929 | 24 | 0.005129 | 82.484985 | 0.687877 | 0.129446 | 0.525199 | 55.62 |
| 02931 | 23 | 0.007339 | 19.662394 | 0.962394 | 0.272423 | 0.680998 | 48.25 |
| 02936 | 29 | 0.004814 | 15.419797 | 0.534346 | 0.329376 | 0.596188 | 51.92 |
| 02948 | 22 | 0.004891 | 34.796799 | 0.684280 | 0.293134 | 0.839252 | 41.26 |
| 02950 | 21 | 0.004171 | 13.838808 | 0.561691 | 0.224578 | 0.540063 | 57.11 |
| 02958 | 27 | 0.003859 | 100.726782 | 0.809206 | 0.104459 | 0.726818 | 53.00 |
| 02981 | 23 | 0.005848 | 43.515326 | 0.928493 | 0.205510 | 0.765026 | 52.45 |
| 03001 | 21 | 0.005190 | 50.777623 | 0.447348 | 0.265073 | 0.843548 | 30.50 |
| 03011 | 20 | 0.006645 | 58.805910 | 1.231528 | 0.113982 | 0.482517 | 32.65 |
| 03012 | 26 | 0.005819 | 17.331492 | 0.851150 | 0.268281 | 0.564565 | 54.87 |
| 03023 | 20 | 0.006590 | 12.219922 | 1.527878 | 0.180129 | 0.688015 | 52.88 |
| 03024 | 22 | 0.004445 | 21.951380 | 0.492151 | 0.382906 | 0.805874 | 52.42 |
| 03027 | 21 | 0.007933 | 38.845084 | 0.716641 | 0.307913 | 0.820641 | 43.50 |
| 03032 | 22 | 0.004159 | 25.872808 | 0.454225 | 0.336600 | 0.542040 | 63.58 |
| 03034 | 24 | 0.005983 | 27.275920 | 1.016562 | 0.221255 | 0.814262 | 56.24 |
| 03037 | 21 | 0.006676 | 30.614345 | 0.961493 | 0.269521 | 0.702516 | 49.72 |
| 03073 | 24 | 0.004908 | 27.079233 | 0.612963 | 0.295619 | 0.688032 | 49.90 |
| 03082 | 22 | 0.008086 | 26.885496 | 0.737327 | 0.353321 | 0.726001 | 53.89 |
| 03083 | 21 | 0.005138 | 46.488419 | 1.283593 | 0.138445 | 0.760548 | 42.28 |
| 03085 | 26 | 0.004235 | 24.324359 | 0.704885 | 0.278259 | 0.902165 | 48.74 |
| 03091 | 24 | 0.009096 | 35.507756 | 1.071163 | 0.220421 | 0.738675 | 49.48 |
| 03098 | 28 | 0.004050 | 19.766471 | 0.294247 | 0.494345 | 0.839933 | 50.36 |
| 03101 | 22 | 0.007418 | 15.995685 | 0.827881 | 0.344926 | 0.549489 | 60.47 |
| 03105 | 26 | 0.004631 | 17.019430 | 0.676387 | 0.289942 | 0.569554 | 66.70 |
| 03115 | 26 | 0.005796 | 17.402412 | 0.800278 | 0.201128 | 0.663566 | 48.74 |
| 03123 | 21 | 0.006767 | 22.144741 | 0.777087 | 0.348964 | 0.813180 | 48.17 |
| 03126 | 22 | 0.007009 | 24.368477 | 0.523836 | 0.352602 | 0.763063 | 39.37 |
| 03144 | 25 | 0.007552 | 20.635435 | 1.455598 | 0.250335 | 0.800326 | 55.09 |
| 03146 | 23 | 0.004452 | 26.037615 | 0.413024 | 0.365085 | 0.765364 | 44.55 |
| 03156 | 27 | 0.006767 | 40.286453 | 0.452914 | 0.203119 | 0.401567 | 24.13 |
| 03158 | 21 | 0.003352 | 106.873655 | 0.425388 | 0.135464 | 0.803106 | 44.28 |
| 03168 | 26 | 0.006015 | 43.021706 | 1.171977 | 0.179824 | 0.775515 | 51.61 |
| 03205 | 22 | 0.004809 | 18.298330 | 0.352220 | 0.456540 | 0.851465 | 51.16 |
| 03209 | 25 | 0.003760 | 100.577338 | 0.488331 | 0.156826 | 0.815131 | 36.50 |
| 03220 | 21 | 0.005219 | 40.743140 | 0.951334 | 0.250009 | 0.848458 | 57.06 |
| 03231 | 23 | 0.006852 | 22.719156 | 1.407610 | 0.197534 | 0.567774 | 59.45 |
| 03242 | 24 | 0.006596 | 36.012286 | 0.787954 | 0.199394 | 0.703353 | 39.90 |
| 03244 | 27 | 0.007215 | 10.799191 | 0.893819 | 0.216392 | 0.268495 | 68.88 |
| 03261 | 24 | 0.004617 | 28.681124 | 0.630323 | 0.225638 | 0.726292 | 36.38 |
| 03263 | 22 | 0.006845 | 15.209779 | 1.000533 | 0.256952 | 0.604849 | 63.16 |
| 03311 | 22 | 0.005086 | 42.796228 | 0.958257 | 0.192107 | 0.708007 | 52.58 |
| 03332 | 22 | 0.004800 | 17.798170 | 0.769732 | 0.313654 | 0.746962 | 60.05 |
| 03353 | 24 | 0.006433 | 19.288188 | 0.950674 | 0.270281 | 0.643242 | 61.29 |
| 03363 | 23 | 0.006304 | 37.085984 | 0.323341 | 0.425133 | 0.717073 | 29.85 |
| 03366 | 24 | 0.004504 | 16.213097 | 0.609754 | 0.340277 | 0.846328 | 74.14 |
| 03368 | 29 | 0.006369 | 33.295276 | 1.242192 | 0.178000 | 0.663170 | 39.85 |
| 03392 | 24 | 0.006088 | 23.335474 | 1.184860 | 0.260830 | 0.802896 | 65.86 |
| 03400 | 23 | 0.003722 | 25.652192 | 0.332614 | 0.474037 | 0.834839 | 64.85 |
| 03409 | 22 | 0.006255 | 28.475952 | 0.799061 | 0.322821 | 0.804558 | 53.05 |
| 03416 | 21 | 0.003810 | 17.428437 | 0.412605 | 0.380600 | 0.897955 | 55.94 |
| 03423 | 29 | 0.007231 | 27.770447 | 1.461232 | 0.187587 | 0.827314 | 47.50 |
| 03438 | 24 | 0.003863 | 55.622184 | 0.401182 | 0.300301 | 0.943649 | 39.10 |
| 03442 | 24 | 0.005721 | 23.533786 | 1.157355 | 0.153606 | 0.628112 | 66.67 |
| 03452 | 21 | 0.005419 | 14.580127 | 0.757804 | 0.322994 | 0.819203 | 52.00 |
| 03458 | 24 | 0.004746 | 27.549080 | 1.047460 | 0.263517 | 0.759602 | 66.71 |
| 03462 | 21 | 0.005229 | 31.870173 | 0.611553 | 0.300789 | 0.732534 | 69.17 |
| 03474 | 21 | 0.004390 | 36.732785 | 0.474658 | 0.387845 | 0.935871 | 44.17 |
| 03494 | 24 | 0.007004 | 9.489859 | 1.273655 | 0.257186 | 0.753366 | 70.00 |
| 03500 | 24 | 0.004867 | 42.242017 | 0.463274 | 0.277635 | 0.681887 | 50.95 |
| 03512 | 26 | 0.005396 | 36.717178 | 1.354507 | 0.157135 | 0.781015 | 50.65 |
| 03529 | 22 | 0.004895 | 61.537168 | 1.038660 | 0.118847 | 0.521658 | 32.11 |
| 03531 | 21 | 0.006614 | 16.563453 | 0.954437 | 0.339835 | 0.707572 | 58.11 |
| 03546 | 20 | 0.006870 | 30.971379 | 1.184574 | 0.212294 | 0.772673 | 50.00 |
| 03556 | 23 | 0.004957 | 20.353272 | 0.540743 | 0.221570 | 0.461070 | 47.85 |
| 03572 | 26 | 0.006612 | 23.391399 | 0.865558 | 0.243761 | 0.705123 | 44.17 |
| 03574 | 22 | 0.003900 | 8.944534 | 0.727744 | 0.356832 | 0.725933 | 65.53 |
| 03580 | 28 | 0.005432 | 47.351330 | 0.845024 | 0.149885 | 0.578589 | 49.88 |
| 03606 | 23 | 0.005030 | 21.149709 | 1.161385 | 0.226090 | 0.857227 | 71.90 |
| 03615 | 21 | 0.004114 | 78.042350 | 0.795149 | 0.139008 | 0.772403 | 43.61 |
| 03616 | 21 | 0.005238 | 30.243715 | 0.649627 | 0.163690 | 0.350180 | 60.94 |
| 03630 | 26 | 0.003546 | 71.244895 | 0.330869 | 0.261393 | 0.822730 | 43.09 |
| 03634 | 27 | 0.005685 | 47.114519 | 1.192108 | 0.063537 | 0.233291 | 46.54 |
| 03642 | 20 | 0.007630 | 58.795740 | 0.967544 | 0.142056 | 0.602561 | 46.71 |
| 03647 | 24 | 0.004354 | 177.173952 | 1.153245 | 0.044755 | 0.788879 | 52.33 |
| 03661 | 29 | 0.005428 | 24.107387 | 0.860325 | 0.303018 | 0.874807 | 63.77 |
| 03662 | 24 | 0.005433 | 64.522295 | 0.992498 | 0.121632 | 0.499903 | 49.24 |
| 03667 | 28 | 0.005289 | 14.737196 | 0.861094 | 0.249622 | 0.744402 | 62.68 |
| 03682 | 22 | 0.007232 | 16.455995 | 0.939323 | 0.215776 | 0.357326 | 60.32 |
| 03683 | 25 | 0.005424 | 23.826593 | 1.042934 | 0.250728 | 0.763029 | 64.05 |
| 03688 | 22 | 0.003359 | 31.788104 | 0.354482 | 0.167381 | 0.527596 | 47.68 |
| 03693 | 24 | 0.006500 | 15.966451 | 0.724760 | 0.082950 | 0.363190 | 41.48 |
| 03697 | 27 | 0.004981 | 26.490525 | 0.949674 | 0.283806 | 0.920224 | 63.46 |
| 03699 | 26 | 0.007523 | 45.794426 | 0.816325 | 0.215840 | 0.796409 | 38.09 |
| 03708 | 19 | 0.007379 | 35.881530 | 0.740014 | 0.261138 | 0.673765 | 39.31 |
| 03739 | 25 | 0.007520 | 17.073788 | 1.216148 | 0.270155 | 0.702715 | 65.36 |
| 03742 | 21 | 0.005257 | 79.628434 | 0.649286 | 0.219875 | 0.851151 | 34.72 |
| 03743 | 21 | 0.005767 | 20.657299 | 0.953505 | 0.245293 | 0.712505 | 58.44 |
| 03746 | 23 | 0.003922 | 89.262443 | 0.220741 | 0.356089 | 0.925181 | 41.45 |
| 03757 | 24 | 0.005292 | 21.828580 | 0.628820 | 0.294686 | 0.575021 | 60.48 |
| 03759 | 23 | 0.003939 | 68.920125 | 0.632231 | 0.188165 | 0.741020 | 44.65 |
| 03761 | 22 | 0.006091 | 19.635730 | 0.625385 | 0.311415 | 0.780954 | 47.89 |
| 03782 | 23 | 0.006917 | 12.143138 | 1.002407 | 0.306226 | 0.578304 | 73.30 |
| 03796 | 25 | 0.005076 | 17.796489 | 0.566043 | 0.383047 | 0.767865 | 38.68 |
| 03854 | 27 | 0.005211 | 12.336562 | 0.950042 | 0.278214 | 0.735495 | 69.29 |
| 03896 | 22 | 0.009041 | 16.364740 | 1.722065 | 0.187031 | 0.574876 | 51.37 |
| 03906 | 21 | 0.005976 | 12.408929 | 1.351670 | 0.266709 | 0.646335 | 61.39 |
| 03919 | 21 | 0.006624 | 16.014552 | 1.060828 | 0.250290 | 0.630264 | 46.78 |
| 03932 | 26 | 0.002419 | 110.310950 | 0.331478 | 0.159711 | 0.919178 | 25.87 |
| 03940 | 20 | 0.005530 | 18.423316 | 0.931080 | 0.285000 | 0.594084 | 59.35 |
| 03944 | 22 | 0.004873 | 56.371875 | 0.769902 | 0.074806 | 0.241020 | 51.37 |
| 03998 | 26 | 0.003604 | 48.543792 | 0.388329 | 0.203487 | 0.804151 | 47.48 |
| 04008 | 26 | 0.005565 | 48.529169 | 1.449427 | 0.107008 | 0.558897 | 56.78 |
| 04016 | 20 | 0.005145 | 25.152561 | 0.573855 | 0.346451 | 0.792979 | 48.35 |
| 04035 | 21 | 0.004524 | 16.611927 | 0.440750 | 0.367166 | 0.517478 | 42.67 |
| 04036 | 21 | 0.010148 | 35.892083 | 1.124026 | 0.209747 | 0.697190 | 53.33 |
| 04037 | 27 | 0.004156 | 34.996032 | 0.609567 | 0.259269 | 0.634725 | 57.46 |
| 04038 | 25 | 0.005724 | 33.270496 | 1.085474 | 0.185272 | 0.640982 | 52.18 |
| 04045 | 20 | 0.007500 | 182.264478 | 2.190036 | 0.025487 | 0.783564 | 31.06 |
| 04049 | 24 | 0.005267 | 70.061546 | 1.066691 | 0.128599 | 0.801170 | 41.81 |
| 04052 | 22 | 0.006927 | 54.005337 | 1.019983 | 0.130565 | 0.557683 | 42.21 |
| 04061 | 23 | 0.004822 | 14.751964 | 1.165430 | 0.240850 | 0.850036 | 62.65 |
| 04070 | 24 | 0.006758 | 42.828594 | 0.825402 | 0.243872 | 0.663749 | 50.62 |
| 04075 | 23 | 0.004574 | 54.314373 | 0.669700 | 0.197644 | 0.720697 | 47.55 |
| 04080 | 21 | 0.006414 | 19.321267 | 0.433938 | 0.393865 | 0.679365 | 42.06 |
| 04110 | 23 | 0.007743 | 28.135257 | 1.376986 | 0.203872 | 0.677982 | 59.35 |
| 04111 | 23 | 0.006596 | 18.419844 | 0.753579 | 0.256364 | 0.765736 | 47.45 |
| 04128 | 23 | 0.008952 | 88.411859 | 0.830039 | 0.141605 | 0.534399 | 30.45 |
| 04137 | 23 | 0.006574 | 83.337700 | 1.080322 | 0.092802 | 0.627693 | 48.35 |
| 04140 | 22 | 0.004595 | 71.711896 | 0.409065 | 0.151631 | 0.499290 | 32.89 |
| 04144 | 32 | 0.004141 | 22.364985 | 0.642670 | 0.309416 | 0.756892 | 60.66 |
| 04145 | 23 | 0.003657 | 71.692103 | 0.501768 | 0.243793 | 0.891344 | 37.00 |
| 04157 | 26 | 0.004623 | 22.850584 | 0.411141 | 0.405537 | 0.680856 | 50.43 |
| 04173 | 23 | 0.006248 | 26.379601 | 1.415048 | 0.190879 | 0.663873 | 64.15 |
| 04175 | 22 | 0.010623 | 33.345931 | 1.512944 | 0.157644 | 0.685590 | 48.26 |
| 04188 | 20 | 0.004750 | 23.712852 | 0.591824 | 0.376925 | 0.868895 | 54.94 |
| 04195 | 22 | 0.009286 | 11.278207 | 1.371865 | 0.249408 | 0.653434 | 48.58 |
| 04205 | 23 | 0.005417 | 23.190157 | 0.701584 | 0.333897 | 0.660030 | 58.80 |
| 04211 | 22 | 0.005877 | 39.913227 | 0.533577 | 0.229573 | 0.594832 | 43.00 |
| 04214 | 30 | 0.004610 | 21.554119 | 0.852397 | 0.215570 | 0.685747 | 68.63 |
| 04225 | 20 | 0.010025 | 33.225682 | 0.856914 | 0.203336 | 0.436543 | 30.06 |
| 04235 | 25 | 0.004560 | 28.258696 | 0.551256 | 0.336754 | 0.842174 | 72.45 |
| 04237 | 25 | 0.006088 | 67.981869 | 0.666224 | 0.176683 | 0.729876 | 46.45 |
| 04252 | 20 | 0.005945 | 153.407759 | 1.542482 | 0.005159 | 0.029891 | 43.06 |
| 04265 | 26 | 0.005058 | 21.986945 | 0.867679 | 0.293280 | 0.838379 | 36.30 |
| 04276 | 24 | 0.004688 | 22.744898 | 0.419143 | 0.351485 | 0.662827 | 56.00 |
| 04283 | 29 | 0.005048 | 23.574026 | 0.945462 | 0.228400 | 0.714793 | 64.77 |
| 04284 | 23 | 0.005226 | 17.539157 | 0.738592 | 0.335950 | 0.649627 | 47.00 |
| 04316 | 27 | 0.005267 | 19.540073 | 1.019576 | 0.272392 | 0.682461 | 68.21 |
| 04327 | 22 | 0.004018 | 17.527708 | 0.622099 | 0.327076 | 0.786126 | 50.74 |
| 04340 | 26 | 0.006446 | 22.104397 | 1.119062 | 0.209293 | 0.603116 | 57.96 |
| 04342 | 20 | 0.009005 | 28.552256 | 1.292435 | 0.143291 | 0.387322 | 41.88 |
| 04343 | 27 | 0.006407 | 31.127472 | 1.157633 | 0.202981 | 0.743141 | 55.67 |
| 04344 | 25 | 0.005388 | 13.263678 | 0.537348 | 0.406288 | 0.638404 | 62.50 |
| 04345 | 27 | 0.007115 | 34.222914 | 0.811236 | 0.172542 | 0.490485 | 49.75 |
| 04365 | 26 | 0.005292 | 47.234226 | 0.876760 | 0.175401 | 0.627186 | 45.74 |
| 04367 | 24 | 0.006558 | 9.937374 | 1.189566 | 0.261576 | 0.608820 | 53.00 |
| 04372 | 24 | 0.004404 | 32.619209 | 0.668265 | 0.302971 | 0.652209 | 56.95 |
| 04423 | 22 | 0.010123 | 14.921552 | 1.669886 | 0.181223 | 0.528989 | 54.63 |
| 04424 | 23 | 0.005443 | 54.362935 | 1.169641 | 0.138949 | 0.638193 | 51.30 |
| 04468 | 20 | 0.005215 | 46.733251 | 0.845468 | 0.152158 | 0.524405 | 40.12 |
| 04488 | 22 | 0.006023 | 25.335367 | 0.581491 | 0.286965 | 0.747663 | 42.95 |
| 04513 | 24 | 0.008175 | 14.785689 | 1.392218 | 0.198256 | 0.725142 | 52.86 |
| 04533 | 27 | 0.006074 | 36.959322 | 0.813582 | 0.221690 | 0.716876 | 43.38 |
| 04537 | 21 | 0.005224 | 34.997451 | 0.579602 | 0.313235 | 0.881327 | 55.56 |
| 04542 | 20 | 0.008340 | 27.267490 | 0.910264 | 0.262265 | 0.801868 | 50.00 |
| 04558 | 20 | 0.005640 | 29.910692 | 0.750376 | 0.274424 | 0.651202 | 52.06 |
| 04567 | 23 | 0.007648 | 25.278345 | 1.182277 | 0.210217 | 0.526847 | 36.80 |
| 04572 | 20 | 0.005840 | 30.060147 | 0.828577 | 0.285023 | 0.820874 | 45.65 |
| 04574 | 21 | 0.005710 | 21.785388 | 0.672282 | 0.335807 | 0.600833 | 64.17 |
| 04581 | 20 | 0.005295 | 102.457290 | 0.697276 | 0.173606 | 0.882083 | 35.82 |
| 04628 | 23 | 0.006561 | 16.895684 | 0.893213 | 0.193343 | 0.350261 | 63.80 |
| 04633 | 22 | 0.008132 | 14.123938 | 1.024135 | 0.312691 | 0.772823 | 50.63 |
| 04662 | 25 | 0.006988 | 24.393629 | 1.417219 | 0.167241 | 0.660367 | 63.91 |
| 04668 | 25 | 0.004944 | 11.184527 | 0.543019 | 0.396767 | 0.781902 | 74.36 |
| 04678 | 26 | 0.004277 | 93.684526 | 0.444181 | 0.241400 | 0.914676 | 45.30 |
| 04693 | 26 | 0.005919 | 20.207365 | 0.856834 | 0.324411 | 0.871235 | 64.48 |
| 04697 | 20 | 0.004625 | 30.529312 | 1.022813 | 0.199085 | 0.757356 | 55.53 |
| 04704 | 25 | 0.005080 | 49.441195 | 0.445149 | 0.315506 | 0.842978 | 46.68 |
| 04727 | 29 | 0.004469 | 15.669215 | 0.689945 | 0.229851 | 0.670900 | 51.08 |
| 04755 | 26 | 0.007450 | 46.575749 | 1.157096 | 0.180684 | 0.631944 | 40.48 |
| 04761 | 27 | 0.008337 | 39.903005 | 1.399494 | 0.114419 | 0.535596 | 40.58 |
| 04767 | 23 | 0.005170 | 19.202221 | 0.517895 | 0.312194 | 0.555313 | 48.95 |
| 04775 | 23 | 0.003117 | 16.553706 | 0.447343 | 0.408962 | 0.684775 | 70.15 |
| 04782 | 20 | 0.004475 | 50.285981 | 0.436289 | 0.388043 | 0.944382 | 38.65 |
| 04787 | 23 | 0.004361 | 24.017108 | 0.357513 | 0.410772 | 0.707785 | 63.70 |
| 04789 | 22 | 0.008091 | 24.529712 | 1.283529 | 0.217916 | 0.836910 | 42.63 |
| 04792 | 23 | 0.007822 | 15.395293 | 0.571134 | 0.469849 | 0.917888 | 51.20 |
| 04797 | 20 | 0.009085 | 31.417206 | 1.767758 | 0.133168 | 0.574733 | 33.82 |
| 04822 | 25 | 0.006340 | 37.394899 | 0.894382 | 0.203341 | 0.644247 | 53.86 |
| 04824 | 22 | 0.005964 | 58.146476 | 0.986632 | 0.159296 | 0.716163 | 32.26 |
| 04863 | 21 | 0.004619 | 29.186905 | 0.590735 | 0.290954 | 0.792373 | 33.44 |
| 04867 | 27 | 0.009952 | 18.803328 | 1.701394 | 0.234438 | 0.826558 | 55.54 |
| 04901 | 25 | 0.007480 | 78.502156 | 0.653480 | 0.145304 | 0.484874 | 29.64 |
| 04905 | 25 | 0.007244 | 27.791895 | 0.961238 | 0.272677 | 0.834666 | 52.14 |
| 04950 | 26 | 0.006215 | 25.888743 | 0.712341 | 0.340883 | 0.769583 | 63.52 |
| 04955 | 20 | 0.005140 | 17.258519 | 0.659519 | 0.263412 | 0.816642 | 53.82 |
| 04974 | 23 | 0.005926 | 80.170892 | 0.748592 | 0.194283 | 0.787835 | 51.60 |
| 04986 | 27 | 0.005781 | 48.074938 | 0.852270 | 0.188622 | 0.668656 | 47.92 |
| 04992 | 26 | 0.005038 | 15.348251 | 0.793605 | 0.313764 | 0.731286 | 53.74 |
| 04995 | 20 | 0.004495 | 64.846516 | 0.699040 | 0.183067 | 0.720564 | 48.59 |
| 04996 | 22 | 0.003818 | 88.125160 | 0.628943 | 0.160549 | 0.808336 | 34.89 |
| 05004 | 23 | 0.003187 | 112.338812 | 0.478154 | 0.072936 | 0.344992 | 34.30 |
| 05017 | 25 | 0.006084 | 30.321302 | 0.964342 | 0.214316 | 0.643641 | 51.68 |
| 05026 | 23 | 0.004404 | 24.504480 | 0.567677 | 0.352885 | 0.785875 | 68.80 |
| 05027 | 28 | 0.003936 | 85.384052 | 0.483401 | 0.265383 | 0.897973 | 42.80 |
| 05031 | 22 | 0.006259 | 14.893570 | 1.243142 | 0.188719 | 0.480189 | 56.63 |
| 05041 | 27 | 0.003441 | 27.884472 | 0.280231 | 0.367219 | 0.814302 | 57.33 |
| 05044 | 26 | 0.005300 | 23.536072 | 0.706012 | 0.271380 | 0.738375 | 56.09 |
| 05045 | 22 | 0.006195 | 34.376359 | 0.666778 | 0.351878 | 0.899549 | 37.53 |
| 05056 | 22 | 0.004595 | 14.978023 | 0.819660 | 0.329304 | 0.750975 | 70.21 |
| 05062 | 22 | 0.005550 | 20.478803 | 0.955175 | 0.264176 | 0.814076 | 51.84 |
| 05073 | 22 | 0.004823 | 11.249364 | 0.944208 | 0.305525 | 0.763591 | 68.26 |
| 05122 | 21 | 0.006481 | 70.452947 | 1.211354 | 0.132649 | 0.740148 | 31.50 |
| 05157 | 21 | 0.005524 | 18.755242 | 0.714105 | 0.266709 | 0.699242 | 55.33 |
| 05172 | 20 | 0.007645 | 24.145455 | 1.402651 | 0.164189 | 0.476063 | 36.12 |
| 05181 | 28 | 0.003339 | 91.263524 | 0.521352 | 0.215463 | 0.886677 | 37.96 |
| 05209 | 20 | 0.004350 | 42.897749 | 0.518262 | 0.295452 | 0.888475 | 55.47 |
| 05253 | 26 | 0.004781 | 127.700831 | 2.112055 | 0.039854 | 0.846668 | 22.48 |
| 05271 | 24 | 0.006104 | 57.774500 | 0.809658 | 0.160088 | 0.652087 | 46.81 |
| 05314 | 27 | 0.007500 | 34.599798 | 1.268645 | 0.156911 | 0.613473 | 47.54 |
| 05324 | 20 | 0.006265 | 31.475114 | 0.713578 | 0.299818 | 0.769449 | 42.35 |
| 05330 | 26 | 0.005131 | 14.030199 | 1.174899 | 0.227824 | 0.698946 | 62.70 |
| 05336 | 22 | 0.005805 | 19.740183 | 0.924991 | 0.287859 | 0.724825 | 50.05 |
| 05337 | 20 | 0.005400 | 70.733401 | 0.671060 | 0.118590 | 0.628353 | 37.35 |
| 05352 | 25 | 0.005296 | 42.863876 | 0.652065 | 0.150737 | 0.416534 | 53.64 |
| 05360 | 24 | 0.004992 | 24.265674 | 0.539953 | 0.440344 | 0.852107 | 52.90 |
| 05376 | 23 | 0.006583 | 10.309634 | 1.268966 | 0.250973 | 0.579440 | 70.00 |
| 05386 | 22 | 0.003795 | 65.633336 | 0.343455 | 0.401456 | 0.939836 | 33.32 |
| 05400 | 26 | 0.006512 | 34.692708 | 1.444137 | 0.124391 | 0.462075 | 54.61 |
| 05401 | 22 | 0.005373 | 12.997536 | 0.749402 | 0.221742 | 0.410090 | 72.42 |
| 05424 | 21 | 0.007314 | 43.576851 | 0.664112 | 0.212456 | 0.489001 | 38.28 |
| 05432 | 29 | 0.003490 | 55.815584 | 0.449312 | 0.264803 | 0.892578 | 48.23 |
| 05450 | 23 | 0.006317 | 19.070333 | 0.898821 | 0.246172 | 0.588005 | 49.45 |
| 05454 | 22 | 0.005632 | 17.933365 | 0.974444 | 0.268672 | 0.633270 | 46.00 |
| 05461 | 22 | 0.008086 | 21.488802 | 1.532026 | 0.141950 | 0.478100 | 48.37 |
| 05471 | 22 | 0.007586 | 21.053264 | 1.319797 | 0.179500 | 0.763555 | 49.79 |
| 05484 | 25 | 0.005708 | 17.490811 | 0.851605 | 0.194519 | 0.427875 | 57.27 |
| 05493 | 24 | 0.004188 | 25.136923 | 0.609857 | 0.323917 | 0.834564 | 52.67 |
| 05494 | 26 | 0.004600 | 55.344919 | 0.726737 | 0.203897 | 0.793642 | 51.61 |
| 05498 | 23 | 0.004374 | 17.950032 | 0.527770 | 0.348836 | 0.696289 | 70.10 |
| 05511 | 21 | 0.004076 | 73.245379 | 0.646479 | 0.213167 | 0.833951 | 57.89 |
| 05532 | 25 | 0.006128 | 35.056889 | 1.538592 | 0.137796 | 0.570086 | 47.32 |
| 05533 | 21 | 0.008224 | 20.967750 | 0.704866 | 0.315014 | 0.611626 | 55.06 |
| 05547 | 20 | 0.007815 | 26.300561 | 1.623603 | 0.140313 | 0.606515 | 46.24 |
| 05552 | 24 | 0.007233 | 18.814895 | 1.220350 | 0.196238 | 0.671266 | 44.43 |
| 05608 | 24 | 0.004425 | 70.123999 | 0.416632 | 0.226717 | 0.771134 | 41.24 |
| 05619 | 25 | 0.007104 | 14.229124 | 1.081324 | 0.310746 | 0.713753 | 63.32 |
| 05637 | 24 | 0.005542 | 25.605375 | 0.724351 | 0.225103 | 0.737308 | 52.05 |
| 05651 | 23 | 0.004487 | 55.567215 | 0.817686 | 0.170336 | 0.664353 | 48.65 |
| 05672 | 22 | 0.006500 | 14.071735 | 0.562305 | 0.401603 | 0.761283 | 50.58 |
| 05678 | 21 | 0.005952 | 14.853830 | 0.695411 | 0.358429 | 0.687203 | 62.00 |
| 05681 | 21 | 0.006219 | 94.416234 | 1.183361 | 0.074103 | 0.618924 | 28.94 |
| 05690 | 21 | 0.004210 | 18.342222 | 0.470897 | 0.276983 | 0.840544 | 52.17 |
| 05692 | 25 | 0.004832 | 15.662368 | 0.719717 | 0.250303 | 0.664460 | 50.91 |
| 05703 | 20 | 0.007195 | 23.786294 | 0.959328 | 0.260320 | 0.680578 | 55.29 |
| 05713 | 24 | 0.007675 | 20.466517 | 0.889960 | 0.278014 | 0.558538 | 35.33 |
| 05718 | 20 | 0.008245 | 15.036726 | 1.133681 | 0.272156 | 0.625540 | 49.76 |
| 05721 | 24 | 0.005388 | 22.077422 | 1.126391 | 0.253624 | 0.781054 | 66.24 |
| 05724 | 23 | 0.005291 | 16.791522 | 0.777504 | 0.299193 | 0.751699 | 59.65 |
| 05746 | 24 | 0.003883 | 69.483804 | 0.590533 | 0.164787 | 0.636283 | 50.05 |
| 05754 | 27 | 0.004074 | 81.911452 | 0.805205 | 0.155664 | 0.803678 | 53.75 |
| 05769 | 26 | 0.006450 | 23.229633 | 1.254176 | 0.176702 | 0.713733 | 51.57 |
| 05785 | 21 | 0.007119 | 87.486088 | 0.785258 | 0.121783 | 0.719579 | 36.11 |
| 05803 | 22 | 0.009482 | 29.487293 | 1.086360 | 0.222243 | 0.584179 | 44.89 |
| 05862 | 20 | 0.005290 | 11.321689 | 0.602415 | 0.275091 | 0.547139 | 54.53 |
| 05889 | 23 | 0.003270 | 52.066780 | 0.314491 | 0.263997 | 0.744218 | 41.90 |
| 05901 | 24 | 0.005008 | 67.998059 | 0.858522 | 0.090556 | 0.409548 | 37.33 |
| 05916 | 21 | 0.006095 | 10.961074 | 0.697253 | 0.363884 | 0.847732 | 52.33 |
| 05920 | 21 | 0.006310 | 21.631583 | 0.813834 | 0.301897 | 0.758962 | 56.56 |
| 05923 | 26 | 0.005188 | 16.408588 | 0.760484 | 0.264556 | 0.574023 | 63.17 |
| 05933 | 25 | 0.006928 | 41.769624 | 1.146544 | 0.195017 | 0.728171 | 49.77 |
| 05955 | 20 | 0.004085 | 24.916093 | 0.415686 | 0.385400 | 0.622730 | 60.65 |
| 05976 | 26 | 0.007377 | 20.611702 | 0.687137 | 0.325916 | 0.753935 | 46.17 |

Taxa = number of taxa analyzed

RCFV = relative composition frequency variability

LB score = branch length heterogeneity score

Average PD = average patristic distance

Slope = slope of patristic distance versus uncorrected p-distance

R^2^ = Pearson's correlation coefficient of patristic distance versus uncorrected p-distance

Average BS = average bootstrap support in single-gene analysis

**Supplementary Table 5. Sample data for all individuals of *Apodomenia enigmatica* collected.**

| **Code** | **Type status** | **Locality** | **Depth** | **Latitude** | **Longitude** | **COI sequence & notes** |
| --- | --- | --- | --- | --- | --- | --- |
| Ap199.1F | Paratype 1: ZMBN 129501 | Bellingshausen Sea | 430 m | 70° 48.713' S | 92° 31.304' W |  |
| Ap199.2C |  | Bellingshausen Sea | 430 m | 70° 48.713' S | 92° 31.304' W |  |
| Ap199.3C |  | Bellingshausen Sea | 430 m | 70° 48.713' S | 92° 31.304' W | MK404653 |
| Ap200.1F/.2E | Paratype 3: ZMBN 129502 | Bellingshausen Sea | 430 m | 70° 48.713' S | 92° 31.304' W | MK404654 |
| Ap207.1F/.2E | Holotype:  ZMBN 129503 | Wright's Gulf | 506 m | 73° 17.780' S | 129° 11.547' W | MK404651; Sequenced transcriptome |
| Ap219.1E | ZMBN 129504 | Ross Sea | 570 m | 75° 19.778' S | 176° 59.106' W |  |
| Ap225.1F/.2C | Paratype 4: ALMNH 21269 | Ross Sea | 457 m | 76° 28.765' S | 165° 44.266' W | MK404655 |
| Ap233.1E | ALMNH 21271 | Ross Sea | 604 m | 76° 14.716' S | 174° 30.247' E | MK404656 |
| Ap237.1F | Paratype 2: ZMBN 129505 | Ross Sea | 513 m | 74° 41.002' S | 168° 28.003' E |  |
| A2147.1E | Paratype 5: ALMNH 21270 | Amundsen Sea | 699 m | 73° 43.292' S | 103° 37.012' W |  |
| PS96_310R |  | Weddell Sea | 419 m | 74° 39.300' S | 26° 53.530' W | MK404652; Sequenced transcriptome |
